# From Formulas to Functions through Geometry: A Path to Understanding Algebraic Computations

**DOI:** 10.3390/ejihpe11040106

**Published:** 2021-11-19

**Authors:** Alice Barana

**Affiliations:** Department of Molecular Biotechnology and Health Sciences, University of Turin, Via Nizza 52, 10126 Torino, Italy; alice.barana@unito.it

**Keywords:** algebraic formulas, algebraic modelling, interactive technologies, mathematics education, representations

## Abstract

The teaching of algebra at the secondary school level has faced a great revolution during the last 50 years. While previously, it was focused on technicisms and pure syntactic rules, the most modern trends recommend using a functional approach to algebra and giving more prominence to conversions among different representation registers than treatments as simplifications and expansions. Nowadays, the daily practice in teaching algebra is still influenced by the traditional approach, and there is a need to offer teachers examples of activities that can give meaning to algebraic computations. This study proposes a set of interactive activities for eighth grade students, with a functional approach to formulas in a geometric context. The goal of the study is to investigate how similar activities can help students to develop multiple approaches to problems, understand algebraic formulas, and discern which main problems they face. The activities were tested with about 300 students, and qualitative and quantitative data were analyzed to answer the research questions.

## 1. Introduction and Theoretical Framework

### 1.1. Towards a Functional Approach to Teaching Algebra

Algebra has a central role in every field of Mathematics: its concepts, principles, and techniques are essential for operating in all the other branches of the discipline. For this reason, algebra has been at the core of secondary school Mathematics for many years, and there is a common belief that a good mastery of algebraic techniques is required to understand advanced mathematics [1,2]. Therefore, the teaching of algebra has always focused on technicisms and pure syntactic rules, as a set of procedures disconnected both from other mathematical knowledge and from students’ real worlds, with the risk of losing the sense that the symbols convey [3].

In this panorama, Italy is not an exception. Traditionally, algebra is introduced at the end of lower secondary school, usually at grade eight, when often entire months are dedicated to operating with monomials and polynomials, solving pages and pages of technical exercises that many textbooks propose. However, in the national indications for the first cycle of instruction of 2012, there is just one brief reference to algebraic computations among the learning objectives at the end of lower secondary school: “*interpreting, building and transforming formulas which contain letters in order to express relations and properties in a general form*” [4]. This objective is included in the “relation and functions” content stream and not in the “numbers” one, which includes the other objectives linked to computation techniques, such as fractions, powers, and properties of the operations. Thus, it is suggested to introduce formulas and letters, not to solve numeric expressions, but to generalize and build mathematical models. The suggestion of avoiding presenting algebra as a set of rules to be learned by heart is not a novelty of 2012. It can be found in the Ministry Programs of 1979, where the algebra of polynomials is not mentioned. However, instead, they say that *“algebraic manipulations detached from concrete references must not have a prevalent role in teaching as well as in assessment*” [5].

These indications are in line with the trends and findings of the international literature in Mathematics education. In fact, since the 1970s, the algebraic content strand considered for school teaching has been widened to include the concept of function [6,7,8], driven by the conviction that modelling provides both a critical reason and an important strategy for studying algebra [9]. The shift towards a functional approach to teaching and learning algebra brought about greater attention to algebraic language as a representational tool for modelling [10]. Under this new light, the traditionally studied objects, such as symbols, formulas, and equations, assume new meanings, and the interactive technologies help in exploring and conveying them [9].

In this approach, representations play a crucial role. The *Encyclopedia of Mathematics Education [11] defines mathematical representations as “visible or tangible productions—such as diagrams, number lines, graphs, arrangements of concrete objects or manipulatives, physical models, mathematical expressions, formulas and equations, or depictions on the screen of a computer or calculator—that encode, stand for, or embody mathematical ideas or relationships.*” Duval [12] extensively studied the role of representations in Mathematics education, starting from the assumption that mathematical objects are inaccessible to human knowledge, and students can only identify them through semiotic representations. This can hinder their understanding of mathematics when they cannot associate a mathematical object to its several representations. Mathematical reasoning occurs through the coordination of registers via two main processes, namely, treatment and conversion. A treatment is a transformation from a representation into another within the same semiotic register, and a conversion is a transformation between two representations in different semiotic registers [13]. While syntactic rules guide treatments, conversions can be more difficult for students because they require them to associate different and apparently unrelated representations to the same mathematical concept [14]. For this reason, according to Duval [14], conversion is the key cognitive function that guarantees the conceptual acquisition of mathematical objects.

Under this perspective, a functional approach to algebra should help students focus on conversions and reason through different media, linking distinct registers to underline different aspects that the mathematical model intended to describe. However, several studies show that this approach is not firmly rooted in daily teaching practices. Pozio and Bolondi [15] analyzed the answers given by all the Italian eighth grade students in 2018 to an item of the INVALSI tests (national standardized tests yearly administered to all Italian students of grades two, five, eight, ten, and thirteen), asking them to express, algebraically, a geometrical model, namely the perimeter of a trapezoid in which one side’s length is given through a parameter. The results show the students’ great difficulties in formalizing algebraically geometrical concepts and investigating the possible causes, among which teaching practices have a prominent role. This study is an example of how Italian students do not master the conversion processes in grade eight. In another study, based on data from INVALSI tests, Bolondi, Ferretti, and Santi [13] focus on an item of the 2018 tests for grade ten, involving algebraic manipulation, that is, treatments within the same semiotic register. They analyzed the students’ answers, only 30% of which were correct. They concluded that in the absence of meaningful personal activity, the students interpreted the semiotic representations as unrelated objects, thus losing the connection with the original mathematical concept, even when they could rely on precise transformation rules. A recent study by Bolondi and Ferretti [16] sheds light on the fact that Italian students even seem to lose algebraic skills from grade eight to grade ten, after two years of Mathematics mainly rooted in algebraic computations. This suggests that the usual teaching practices, based on the simplification of algebraic expressions and resolution of equations, are not the most effective to develop a deep understanding of algebraic formulas. Similarly, other studies show that, without appropriately designed activities, the risk of losing the ability to integrate different kinds of reasoning, while dealing with mathematical formulas and functions, is common [17,18].

### 1.2. Geometry as a Context to Work with Algebra

From the literature, we can find several suggestions for teachers and researchers to present algebra through a functional perspective. For instance, geometry can be a relevant context to work with algebraic formulas. In geometry, the use of symbols and formulas to express relations is common and students are used to this practice since primary school, when they face formulas for perimeter and area of the simplest shapes. Moreover, Cartesian geometry involves correspondences between algebraic objects (such as variables, equations, and their solutions) and geometrical ones (such as lines, points, and conic sections). Several scholars suggest using algebraic thinking in geometry to favor the understanding of variables as quantities to be related rather than symbols to manipulate [19,20,21,22]. Activities of this kind include tasks that ask one to compute the perimeter, area, or volume of a figure whose measures are given through variables [19]. Modifying the variable’s value to enlarge or reduce the size of the figure can help move from symbolic manipulation to functional covariation. Wilkie [20] proposed the opposite task: instead of deducing a formula from a geometric property, in her study, participants had to generate new figures and growing patterns expressing given quadratic functions. The results showed that this kind of task can stimulate creativity, help learners make connections among different representations, and support understanding of algebraic relations through visualization. Garzón e Batuista, in [21], studied tasks involving algebra tiles (i.e., squares and rectangles of different dimensions whose areas are represented by polynomials) using a virtual learning environment. They showed that this kind of mathematical manipulative was effective to develop understanding of polynomials, both in the short and long term. The authors [21] argued that similar tasks encourage relational thinking and promote algebraic reasoning; moreover, the digital environment helped increase motivation.

As shown in [15], through large-scale assessment as mentioned before, students often have difficulties in tackling similar algebraic tasks in geometry context. Dindyal [19] found that the students’ main difficulties in facing this kind of problem were in their understanding of geometric properties and algebraic rules. The previously cited study by Pozio and Bolondi [15] added that, besides misconceptions and formal and procedural errors in both the geometric and algebraic spheres, other problems could derive from the inability to coordinate different forms of representation. The roots of such inability can be found in the usual didactic practices, which tend to focus mainly on manipulative mechanisms in the construction of concepts, giving little relevance to functional and semantic aspects.

### 1.3. Algebra, Problem Solving, and Creativity

Another study [1] compares the effects on the growth of students’ understanding, skills, and problem-solving abilities in algebra of two high school Mathematics curricula—the Core-Plus Mathematics Project (CPMP) and a traditional curriculum. The CPMP was based on a real-life problem-solving approach, leaving space to explore concepts rather than teacher explanations and student practice of routine symbol-manipulation skills, as was a common habit in the traditional curriculum. From the study results, it emerges that the abilities in algebraic manipulation are weakly correlated to problem-solving skills, and the authors argue that it is possible to develop advanced problem-solving skills without innumerable repetitive algebraic manipulation exercises. Moreover, managing conversions between different representation registers proved to be key for success in problem solving. In the CPMP, the sense of connection among representations (such as graphs and expressions) for mathematical concepts (such as that of a linear function) was enabled through a variety of experiences with representational activities and applied problem situations [1].

In this study, following D’Amore [23], we refer to problems as tasks, which involve the use of more rules and notions (some of them could also be in the process of being made clear by students in that very occasion), or the sequence of operations chosen by the student with a strategic, sometimes creative, act. This definition is in line with Pehkonen’s [24] one, who refers to a problem as *“a task situation where the individual is compelled to connect the known information in a way that is new (for him) in order to do the task. If he immediately recognizes the actions needed to do the task, then it will be a routine task for him. Thus, the concept “problem” is bound to time and person”*.

These definitions involve the concept of mathematical creativity, a construct that lacks a commonly accepted definition and is applied to all the domains of Mathematics education [25]. We agree with the framework provided by Leikin and Lev [26], based on Torrance’s work [27], which considers creativity as a construct composed of three main traits: fluency, flexibility, and originality. Fluency refers to the development of multiple mathematical ideas, different answers to a mathematical problem (when they exist), and exploring mathematical situations. Flexibility is shown when students are able to generate new solutions to a mathematical problem when at least one has already been produced. Novelty, or originality, involves the exploration of many solutions and the generation of new ones [27].

Solving mathematical problems in multiple ways is closely related to mathematical creativity [26]. In the literature, there is an open debate about the relation between mathematical ability and creativity [23]. A study by Leikin and Lev [26] shows that fluency and flexibility do not seem much influenced by math ability, while novelty shows a higher correlation with ability. The authors conclude that creativity could be fostered by mathematical instruction. This view is shared by many other scholars, as follows: Pehkonen [24] states that problem solving fosters creativity, and this is one reason why problem-solving activities should be a central practice in school curricula. Similarly, Silver [28] suggested that creativity can be encouraged by the use of multiple solution problems. In particular, Silver argued that creativity-oriented mathematics teaching practices, such as exposing students to problems that allow a class to engender different solutions, is associated with the development of students’ representational and strategic flexibility. Representations, as many authors found, are closely connected to the strategy choices in problem-solving activities, since a particular representation can suggest the use of a certain strategy [29].

### 1.4. The Role of Technologies in Algebraic Modelling Problems

In line with [1], Yerushalmy [9] noted that working on long-term vertical paths with algebraic modelling problems that aim to coordinate different forms of representation, students manage to integrate them and better understand the concepts. In this case, technology plays a leading role, mainly helping students connect the graphic and the symbolic registers. Given her results, Yerushalmy [9] rejected the hypothesis that using graphing technologies or computer algebra systems might prevent students from developing skills such as drawing graphs or manipulating expressions autonomously. She observed that the students who successfully used graphing technologies to solve the tasks completed them just as well even without the software. On the other hand, low achievers who had difficulties solving the tasks with paper and pen had the same problems even with the software’s help. As Huntley et al. [1] observed, graphing technologies and computers make modeling and functions attractive and accessible for students, independently of their prior achievements, aptitudes, and interests. The use of calculating tools also offers students a variety of powerful new learning and problem-solving strategies. Thus, the need for students to acquire a high degree of skills in symbol manipulation diminishes.

### 1.5. Algebra as a Language for Modelling

Malara, Cusi, and Navarra [6] suggested using natural language as a didactic mediator for learning algebra. Analogously to every language, algebra has a syntactic structure and a semantic aspect; therefore, it should be taught as a new language—starting from its meanings and setting them gradually in the syntactic structure. They called this process “algebraic babbling” [6]. The process of translation (from algebra to natural language and vice versa—from one representation to another) is central. Natural language sets up the bases for producing and interpreting representations written in algebraic language. Through natural language, students can illustrate the systems of relations (additive and multiplicative ones at the beginning) among elements in a problem situation, inducing a translation of the process itself into a mathematical sentence. In this way, in the resolution of a problem, attention is shifted from the arithmetic goal of solving it in order to produce a result, to the algebraic one of representing it, its variables, and relations. Under this perspective, algebra becomes a language for modelling.

Many authors have recently suggested starting the learning of algebra early, using the approaches that we presented above, from primary school [6,30,31]. In order to improve the teaching and learning of algebra, Kaput [30] suggested beginning early, as follows: integrating the learning of algebra with that of other disciplines (that is, to apply it); including different forms of algebraic thinking; grounding in the students’ cognitive and linguistic abilities; and encouraging active learning. He distinguishes five different forms of algebraic thinking, as follows:

- generalization and formalization of patterns and constraints;
- syntactically guided manipulation of (opaque) formalisms;
- study of structures, abstracted from computations and relations;
- study of functions, relations, and joint variation;
- cluster of modeling and phenomena-controlling languages.

By using these interacting forms of algebraic thinking, it is possible to move from the abstract and formal algebraic rules to the language for modelling.

### 1.6. Goal of the Paper and Research Questions

From this brief review, we can notice that there is a need for designing innovative activities aiming at developing deep understanding of algebraic objects and rules from the earliest years students meet them. At the same time, teachers need to be made conscious of the limitations of classical textbook exercises and trained to design their own activities [7]. This study presents and discusses the design of a set of digital activities for developing a functional approach to algebra at grade eight, when students, in Italy, begin to approach formulas and symbols. These activities were proposed to 299 students, and the results were analyzed from a qualitative and a quantitative point of view. The goal of this paper is to show how similar activities can contribute to the full understanding of algebra, the development of modelling skills, and creativity in problem solving. In particular, we study whether algebraic thinking problems in a geometric context can be a valid starting point to introduce functions.

The research questions guiding this study are the following:

(RQ1) Could an activity of algebraic thinking in a geometric context help students to develop mathematical creativity?
(RQ2) In what ways can the use of multiple representations support the understanding of algebraic formulas?
(RQ3) What are the main problems faced by students in using algebraic thinking in geometry?

The structure of this paper is as follows: Section 2 (Materials and Methods) is dedicated to the presentation of the digital materials and the design of the teaching experiment; in Section 3 (Results), the results of the experiment are shown; in Section 4 (Discussion) the results are discussed in the light of the aforementioned theoretical framework, and in Section 5 (Conclusions) some conclusions and implications for research and didactics are drawn, respectively.

## 2. Materials and Methods

To answer the research questions, we designed a teaching experiment involving 299 students of grade 8 (13 years old) from 13 classrooms of 6 lower secondary schools in the town of Turin, Italy. The experiment took place in the 2017–2018 school year, from December to June. It was part of a bigger project called “Città Educante” (i.e., Educating City), from which the DELTA (Digital Education for Learning and Teaching Advances) Research Group of the University of Turin was able to obtain important results in the field of automatic formative assessment and digital education in Mathematics [32,33,34]. Here, we will analyze some of the project’s activities from the point of view of the understanding of algebraic formulas.

### The Interactive Activities

For that which concerns this study, an interactive path, concerning formulas and functions, was created in a digital learning environment [35]. The interactive path was made available to students and their teachers, who could use the activities as a basis for their lessons and homework. The activities were mainly used in two different modalities, as follows:

- In the classroom, displayed through the interactive whiteboard (IWB) and asking students to solve them in small groups, with paper and pen or with tablets, if available.
- At home, as homework, to be carried out individually.

Part of the lessons was held in collaboration with a researcher, the author of this paper, who helped the teachers to guide the activities and offered technical support if needed. At the beginning, halfway through, and at the end of the school year, students were asked to fill in some tests with multiple-choice and open-ended questions about the topics covered in the learning path. The tests were aimed at verifying the acquisition of skills and competences in algebraic reasoning and modelling.

The materials were designed using a problem-solving approach [23,24,36,37] and they were proposed through the following:

- interactive worksheets built using an Advanced Computing Environment (ACE), where students could explore the mathematical models and objects, change the value of the variables, and observe the variations in the results [38,39]; or
- automatic assessment activities using an Automatic Assessment System (AAS), particularly suitable for STEM subjects [40,41], designed according to a model for automatic formative assessment and interactive feedback proposed by the DELTA Research Group [42].

All the materials were made available through a Moodle platform, freely accessible by students and their teachers [43].

**Figure 1:**
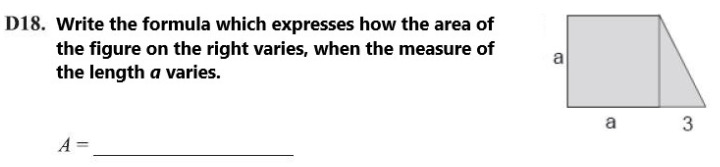
An item from INVALSI tests involving algebraic reasoning on a geometric figure. The text has been translated into English. Adapted from [44].

The path originates from some activities on algebraic computations and manipulation of algebraic formulas, seen in a geometry context. Symbolic computation is a core topic in the Mathematics curriculum of grade 8. Algebra constitutes one of the bases for operating with functions, which will be one of the main objectives of upper secondary Mathematics. Writing a formula to calculate the area, the perimeter, or the volume of a figure whose measures are expressed through letters and numbers is a pretext to analyze—from a geometrical, numerical, graphic, and algebraic perspective—linear, quadratic, and cubic functions. Algebraic thinking in geometry is proposed by several scholars to favor the understanding of variables as quantities to be related, rather than the symbols to manipulate [19,20,21,22]. We developed a set of tasks on symbolic computation taking inspiration from an item of the INVALSI tests for grade 8 of 2009, shown in Figure 1. It asks students to compute the area of a geometrical shape, a right trapezoid, whose measures are given in function of a variable. In order to correctly answer to the task, as a first step students need to find a way to compute the area of the trapezoid. They can recall the formula or decompose it in simpler shapes, with the given figure’s help. As a second step, they need to use algebraic rules to add and multiply algebraic expressions. Synthetizing the obtained formula in a compact form is not requested, with the answer being open. To solve the item, students have to combine geometrical and algebraic reasoning. The relation between geometry and algebra is what makes this task interesting. Measures of geometrical shapes are used to visualize algebraic operations, and it helps confer concreteness to abstract computations. The question was difficult, with 27% of students achieving correct answers. This indicates the students’ low level of flexibility in connecting different areas of mathematical knowledge; nevertheless, the processes that this item involves are very interesting from a didactic point of view.

**Figure 2:**
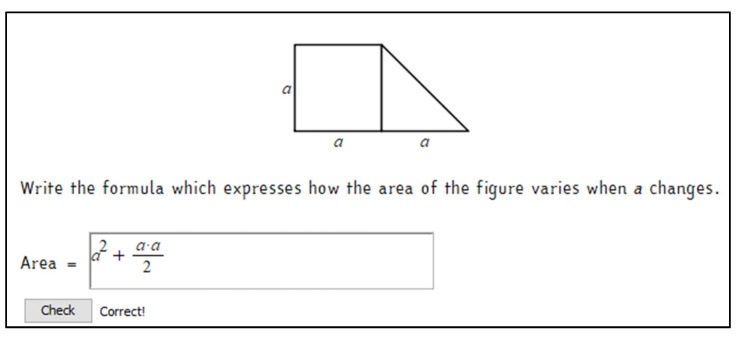
First part of the interactive activity “Formulas and Areas”. The students have to find a formula to express the trapezoid area, write it in the box, and check its correctness. The activity, originally in Italian, has been translated into English for the comprehension of this paper.

**Figure 3:**
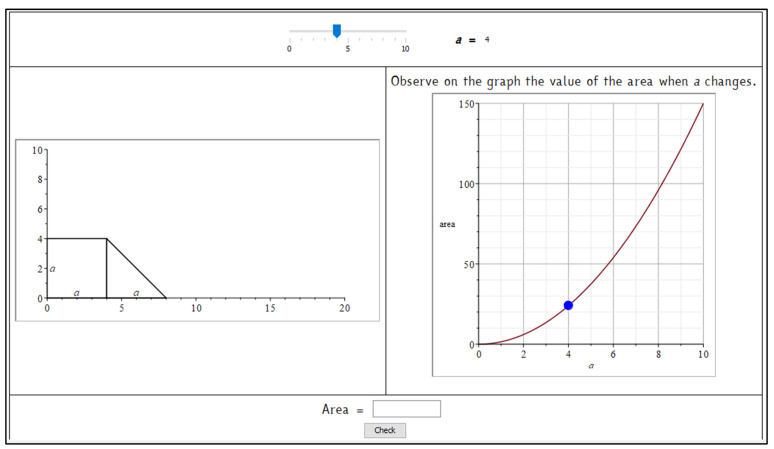
Part of the interactive activity “Formulas and Areas”. Students can move the slider choosing a value for the variable *a* and observe the figure increasing and the point moving along the graph of the function. The activity, originally in Italian, has been translated into English for the comprehension of the paper.

Starting from these considerations, we built a set of tasks with automatic assessment and formative purposes. They were conceived for making algebra less abstract through a geometry contextualization, which could help students visualize symbolic computations. During the classroom activities, they were used as a starting point for the learning path to introduce algebra with a functional approach and use it as a language for modelling. Here, we present the first activity, “Formulas and Areas”, which asks students to write the formula that expresses the area of a trapezoid (Figure 2). The symbolic register is immediately required, associated with the geometric representation. An interactive file, built using Maple ACE, was integrated within the platform (part of which is shown in Figure 3) and served as a support for a classroom discussion about the possible correct formulas and as a guide for discovering the function obtained when the parameter varies. The increasing of the trapezoid measures and, consequently, of its area, is associated with the movement of a point along a curve of the cartesian plane. It corresponds to the numerical results previously computed and collected in a table. Here the numeric, geometric, and graphic registers are associated and jointly vary. The activity was repeated modifying the initial figure and fixing the length of one size (the triangle’s base, like in the original INVALSI item). Other activities were developed through interactive worksheets, automatic formative assessment, and involved non-standard shapes and solid shapes.

In order to answer the research questions, we used the following sources of data:

- observation and notes, taken during the classroom activities by the teachers and the researcher;
- videotapes of the classroom activities;
- students’ answers to specific items of the final test.

In particular, the videotapes were qualitatively analyzed according to the frameworks about creativity and representations presented above. Some episodes were selected among the data collected during the activities for their meaningfulness in relation to the research questions. To confirm the qualitative findings, one item of the final test—the one mainly related to the algebraic skills under analysis—was considered. For this question, we compared the results of our sample with those of the national sample that faced a similar question during the INVALSI tests.

## 3. Results

The “Formulas and Areas” activity was carried out by all the students in our sample in a classroom-based modality with paper and pen. The teacher, along with the researcher, displayed the tasks, one at a time, through the IWB and asked students to solve them in groups. The groups’ conclusions and results were collectively checked through a classroom discussion, supported by the interactive materials. The following schema was used: at the beginning, the main task’s figure was shown through the IWB, and students were asked to find a formula to express its area. The groups of students were encouraged to find as many formulas as they could. The teacher and the researcher passed through the groups to monitor their work and answer their doubts, while a university student was videotaping some chosen groups’ work. When all the groups had found some formulas, all the formulas were shared through the IWB, asking each group to explain how they found them. The equivalence of the different formulas under a geometrical and algebraic perspective was discussed. After this more “algebraic” part, the activity shifted toward a functional interpretation of the formulas. Firstly, the students were asked to choose a unit, assign progressive values to the variable, and draw one figure for each value, computing the related area. In this way, they could observe the figure growing together with its area. Then, the values of the variable and the corresponding areas were collected in a table; these values were used to build the graph of the function. The groups worked autonomously, with each student working on their notebook. In the end, the function was checked through the interactive worksheet at the IWB and collectively discussed.

The activity is an example of a problem with multiple solutions [37]. The context is not from the real-world because adding a real contextualization would feel forced and add an irrelevant difficulty to the problem. However, it allowed the students to find multiple solutions, without suggesting any particular solving strategy. In all the classes, at least 7 or 8 formulas emerged, such as:

- $\frac{\left(a\;+\;a\;+\;a\right)\cdot a}{2}$—seeing the figure as a trapezoid;
- $a^{2}+\frac{a\cdot a}{2}$—interpreting it as the composition of a square and a triangle;
- $3\cdot \frac{a\cdot a}{2}$—splitting the square into two triangles through the diagonal, and interpreting the trapezoid as composed by three identical triangles;
- $2a^{2}-\frac{a\cdot a}{2}$—seeing a rectangle with base 2*a*, minus the upper right triangle;
- $2a^{2}-\frac{1}{4}\left(2a^{2}\right)$—seeing a rectangle to which a quarter of the rectangle itself was removed;
- $\frac{2a\cdot a}{4}\cdot 3$—dividing the rectangle into 4 parts to find a triangle and multiplying it by 3.

**Figure 4:**
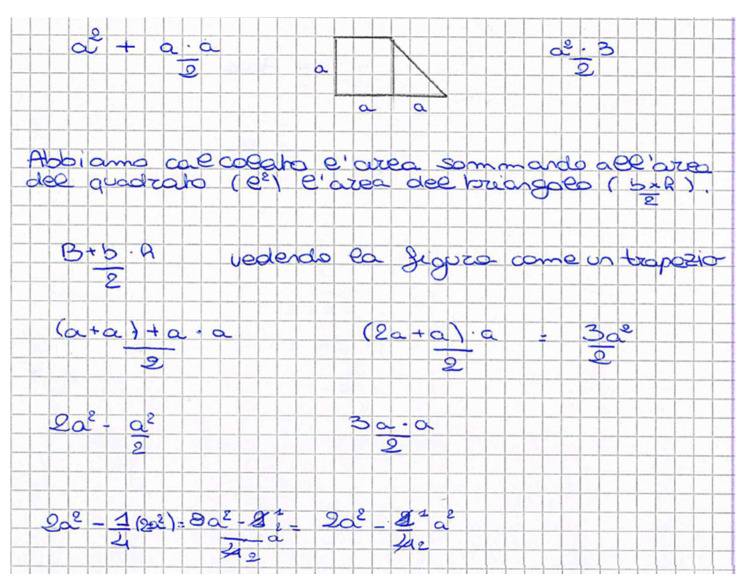
Notes of a group of students working on the formulas for computing the trapezoid area. Some sentences next to the formulas describe the reasoning that was followed to write those formulas.

These are only some examples of the problem’s solution set, as the students gave rein to their creativity and decomposed the figure in many different ways, each one leading to a new formula. Figure 4 shows, as an example, the notebook of a group of students full of different formulas.

**Figure 5:**
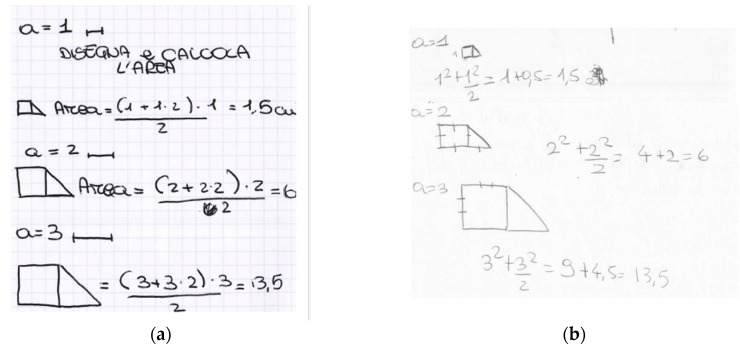
Two students’ computations of the area of the trapezoid. In (**a**), the student used a geometric approach and computed the area using the classic formula for the trapezoid area. In (**b**), the student used an algebraic approach and used the simplified formula to compute the area.

In all the classes, the teacher and the researcher spent some time making students notice that they could solve the problem in many different yet correct ways and that everyone could choose the strategy they preferred. All the solutions were reconducted to the simplified form $\frac{3}{2}\;a^{2}$, not in order to do exercises of symbolic manipulation, but rather to show that all the formulas were equivalent from an algebraic perspective, not only from a geometric perspective. In this way, algebraic treatments were supported by a geometrical interpretation to give them meaning. The algebraic equivalence of the different formulas expressing the same area was not taken for granted by most of the students—they needed to prove it to be sure. Moreover, when they were completing the tables with some values of the variable and the corresponding areas, the teacher and the researcher asked students which of the many found formulas they had been using to compute the area. There were many different answers. Some students used a geometric approach and chose the first formula they found, reflecting their preferred geometric reasoning (often expressing the trapezoid area or the composition square plus triangle). In this way, each time, they followed a classic procedure to compute the area. Others preferred an algebraic approach and chose the simplified formula so that computations were easier. Figure 5 shows two examples of students’ computations of the trapezoid area for several values of the variable. On the left, the student chose a geometric approach and used the classic formula for the trapezoid area to calculate the area. On the right, the student followed algebraic reasoning and used the simplified formula. The preferred representation system influenced the students’ solving strategy. Highlighting the different choices, all of them correct and some more convenient for the computations, was another way to emphasize that problems can be solved in many ways, some more convenient than others. Everyone was allowed to choose their preferred strategy.

In a class of a school attended mainly by high-achieving students, while all the groups were finding formulas for the area of the trapezoid and the researcher was going around the room to observe their work, one of the students called her over. The following conversation took place.

**Excerpt 1**

JUAN: I am solving the task differently. I am trying to solve it using functions.

RESEARCHER: Good! How are you doing?

JUAN: I tried to compute the area for some cases, and I’m plotting them in a graph.

RESEARCHER: Very good! Go on.

After some minutes, the researcher came back to Juan and asked if he managed to find the function.

RESEARCHER: How are you doing with your function?

JUAN: I am doing it, but I do not think it is correct, because the points are not aligned. I think it’s wrong, isn’t it?

In this excerpt, Juan understood the role of *a* as a variable, and he was trying to represent the area graphically without passing through the symbolic representation. The class had already studied functions and their representation in the Cartesian plane. They were used to deal with linear functions, so the fact that the points were not aligned made Juan think that his function was incorrect. However, Juan rapidly understood that the formula could be interpreted as a function and plotted in a graph. He showed a high level of novelty in choosing this strategy, since nobody else in his classroom thought about it as a first approach.

Students also found the same formulas by reasoning in different ways. The geometry context helped them understand that they were all equivalent. To this extent, we report an excerpt of a conversation that happened at another school, during the discussion on the students’ formulas.

**Excerpt 2**

SARA: We found: $a^{2}+\frac{a\cdot a}{2}.$

RESEARCHER: Good! How did you see the figure to write this formula?

SARA: We divided the figure into a square, whose area is, and a triangle, and we computed base times height divided by two, that is

RESEARCHER: Very good! Did anyone find another formula?

MARTA: Yes, we did! Ours is similar, but we thought it differently.

RESEARCHER: What did you think?

MARTA: We thought that the figure is a square plus half the square, so we did

RESEARCHER: Is it the same formula?

MARTA: Yes, because is equal to

TEACHER: Correct! [pointing at the figure at the IWB] You see, guys, computing the area of the triangle, that is *a* times *a* divided by two, is the same that dividing by two the area of the square, that is squared *a* divided by two.

In the previous excerpt, we could notice how the students interpreted the problem in different ways, though finding the same results. Moreover, the correspondence with geometry helped the students understand mathematical equivalences and treatments, even those that often we take for granted. In this case, treatment ($\frac{a^{2}}{2}=\frac{a\cdot a}{2}$) is explained by two conversions from an algebraic register to a geometric register (the area of a triangle and half the area of a square), and the geometrical equivalence, which is more intuitive, justifies the algebraic equivalence. The request to make explicit and collectively discuss all the formulas that students found is a strategy to make them put their formulas into words and use algebra as a language. Moreover, through this and other similar dialogues, students are trained to develop fluency and flexibility in switching among different solving strategies.

In a class of another school, mainly attended by students from lower social classes, the following discussion was registered during the activity about “Formulas and Areas”.

**Excerpt 3**

SANDRO: We found: $2a^{2}-\frac{1}{4}$.

RESEARCHER: Ok. How did you reason?

SANDRO: We considered the rectangle that has as a base the longer base of the trapezoid and as a height its height, and we removed a quarter, which is the upper right triangle.

RESEARCHER: Ok. So, you considered the whole rectangle and said that the trapezoid is three-quarters of the rectangle. Is it correct?

SANDRO: Yes, that’s right.

RESEARCHER: Are you sure that your formula is correct, written like that?

SANDRO: Yes … mmm … I’m not so sure.

RESEARCHER: And you guys? Who thinks that the formula is correct?

CLASS: [Silence, and whispers of students discussing in groups]

PAOLO: No, in my opinion, the formula cannot be correct because it cannot be $\frac{3}{2}a^{2}$, which is the correct formula.

TEACHER: Why not?

PAOLO: Because you cannot do $2a^{2}-\frac{1}{4}$, you cannot subtract it, because you can only subtract similar monomials.

TEACHER: Ok, Paolo made a good point. Do you agree with him?

CLASS: [Whispers of students who do not seem much convinced with Paolo’s idea]

CHIARA: We could try a few times with different numbers.

TEACHER: Yes, Chiara’s idea is good. We can try with a=2 and see if the result is the same. If a=2, what is $\frac{3}{2}a^{2}$?

CLASS: [After some calculations with paper and pen] It’s 6.

TEACHER: Ok. What about $2a^{2}-\frac{1}{4}$?

CLASS: [After some calculations with paper, pen, and calculator] It’s 7.75.

TEACHER: Ok, so it is not the same. Let’s try with 3 and 4.

CLASS: [After working for some minutes] No, they are not the same!

MATTIA: You do not subtract enough things each time!

TEACHER: Why not? What are you subtracting?

MATTIA: A quarter.

TEACHER: And what should you subtract?

MATTIA: One quarter … of the rectangle!

TEACHER: Yes. And how do you write it? A quarter OF the rectangle? How do you express the reference to the rectangle?

ANNALISA: Right, we have to write $2a^{2}-\frac{1}{4}\left(2a^{2}\right)$!

At that point, they were quite convinced of why the functions were not the same. To reinforce the concept, when they drew the function of the area, the teacher asked them to add the function $2a^{2}-\frac{1}{4}$ on the graph. Observing the different trends of the two functions, the students understood the concept.

In the last excerpt, we can see how the discussion involved several registers of representation and various conversions. The original idea for the function comes from geometrical reasoning. Paolo’s doubts were of an algebraic nature, as he considered that the formula does not match the “correct” one, which had previously been discussed. Reasoning using a numerical approach helped students give meaning to the formulas, thus understanding what the problem was with the incorrect one. The conversion into a graphic representation helped them clarify the difference between the correct and the incorrect functions, have a mental picture of the concept, and understand it. Towards the end of the discussion, the translation from natural language to algebraic language was used to focus on a missing part of the sentence, which is the reference to the rectangle. In this episode, the students were engaged in multiple conversions of the same concept, expressed in different registers: natural language, geometry, symbols, numbers, and graphs. The orchestration of the different representations was successful in improving the understanding of a non-trivial concept.

Through the observations during classroom activities and videotape analysis, we collected the most frequent mistakes made by the students when solving the tasks of algebraic modelling in a geometry context. Here, we present some examples showing the most typical mistakes.

In the following exchange, which occurred during group work, Mariana and Federico discussed the area of the trapezoid.

**Excerpt 4**

MARIANA: I have an idea! We can consider this rectangle, you see, and choose only one quarter, then multiply it by 3.

FEDERICO: I can’t follow you. Write here.

MARIANA: Well, we can take this rectangle, its area is 2a·a. Then we divide it by one quarter, so we have only one triangle.

FEDERICO: [Writing] so, $\frac{2a\cdot a}{\frac{1}{4}}$.

MARIANA: Yes, now we can simplify it, and we have $\frac{a\cdot a}{2}$.

FEDERICO: [Writing the simplifications] Yes.

MARIANA: And now we can multiply it by 3, so we have three triangles that are our figure!

FEDERICO: Well done, you are a genius!

In this excerpt, Mariana showed creativity with an original idea, but she committed an error in formalizing it: choosing one quarter became “dividing by one quarter.” Federico did not notice the mistake but continued to follow her reasoning. A second mistake in the simplification restored the correct result. This kind of error derives from misconceptions in working with fractions, which are very common but relate to the basic arithmetic area.

The following excerpt refers to an conversation that occurred during the collective discussion of the formulas for the trapezoid area.

**Excerpt 5**

ALEX: We wrote a+a, that is $a^{2}$, then…

TEACHER: Are you sure?

ALEX: Yes, a+a … oh no, that’s 2a!

TEACHER: Look at the figure. What is a+a?

ALEX: It’s the long base of the trapezoid

TEACHER: And what is $a^{2}$?

ALEX: It’s the area of the square

TEACHER: Could they be the same thing?

Here, Alex made a very typical mistake of an algebraic nature, confusing the position of power and coefficient. The geometric context helped him understand the mistake: the addition is associated with a linear measure, while the square is associated with an area—they have two different natures and cannot be considered as equivalent.

**Excerpt 6**

The class was discussing the formulas resulting from the second part of the activity “Formulas and Functions,” where, in the trapezoid, the horizontal base of the triangle is fixed b=1.

MARCO: We wrote: squared *a* plus square root of squared *a* plus squared *b*, which is *c*.

TEACHER: What do you mean?

MARCO: We computed the area of the square, that is $a^{2}$, then the area of the triangle, using the Pythagorean Theorem, so $\sqrt{a^{2}+b^{2}}=c$.

TEACHER: And what do you find doing $\sqrt{a^{2}+b^{2}}$

MARCO: The hypothenuse.

TEACHER: And how do you compute the area of a triangle?

MARCO: It’s base times height divided by two.

TEACHER: So, where is the hypothenuse?

In this discussion, Marco and his group tried to compute the area of a triangle using the Pythagorean theorem. This mistake, related to the problem’s geometric interpretation, probably derives from the habit of using the Pythagorean theorem when dealing with triangles. It causes an immediate reaction in the student’s mind: every time there is a triangle in a problem, they try to use the Pythagorean theorem, without thinking about whether it makes sense. By reasoning on the formulas’ semantic aspect and the computations’ meaning, students can identify the mistake and correct it.

**Excerpt 7**

The students of a class in another school were working in groups on task involving a new figure. After some difficulties, the class managed to write the correct formula (i.e., 3a+2(a−3)), and they were collectively discussing it with the teacher.

TEACHER: Ok, we found the formula 3a+2(a−3). It is correct. Can we carry out the multiplication?

CLASS: [Silence, thinking about the teacher’s request]

ANDREI: No, we cannot do the multiplication.

TEACHER: Why not?

ANDREI: Because we cannot say how much it is.

The students were destabilized because the result was not a number, as they were used to in geometry tasks. After a lengthy discussion, the teacher managed to convince the class that, even if they were solving a geometry task, they could do algebraic simplifications of the formulas. The habit of studying the various mathematics branches, separately, blocked the students who had difficulty finding and manipulating a formula when dealing with measures and areas. Moreover, as observed by several authors [7], multiplications are usually carried out with numbers to find a new number; here, students find it hard to understand that they can also multiply variables.

**Figure 6:**
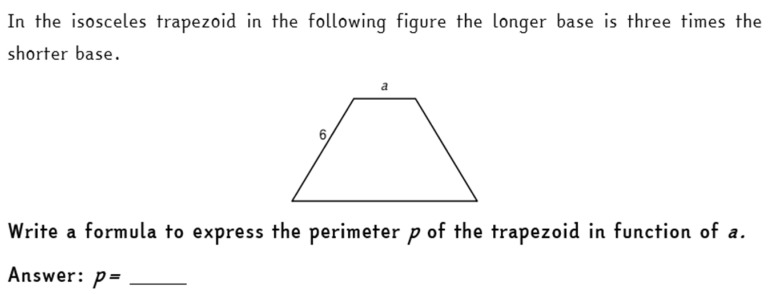
Item of the final test, dealing with algebraic modelling in geometry. The item, originally in Italian, was translated into English for the comprehension of this paper.

We checked if the reasoning promoted through these activities was assimilated and deeply understood by the students through the final test, which students undertook about two months after working on the “Formulas and Functions” activity. The final test included an item of algebraic manipulation in a geometrical context, where students were asked to compute the perimeter of a trapezoid whose sizes were given through variables and constants. The text of the item is shown in Figure 6. This item is similar to an item proposed in the 2018 INVALSI test to all the Italian students of grade 8 (the same item analyzed in [15]). We computed the rate of correct answers to this item and compared them with the rate of correct answers registered by the national sample of the INVALSI test. We found that the students participating in this experimentation registered a high percentage of correct answers (67%). This percentage is clearly higher than the national sample’s one, which only achieved the 34% of correct answers, when they faced a similar question.

## 4. Discussion

Synthetizing the results presented in this paper, we can answer the research questions initially proposed. We can positively answer RQ1, which was “could an activity of algebraic thinking in a geometry context help students to develop mathematical creativity?” In fact, we saw that the students found different solutions to the problem “Formulas and Areas”, interpreting the figure in several different ways: even when they proposed the same formula, often there were different reasonings in the background. In particular, the same group of students found different solutions to the problem, showing fluency and flexibility. Moreover, the different choices of the formula used to substitute the values and compute the figure’s areas showed the heterogeneity of the approaches used to tackle the problem. The modelling process itself was undertaken in different ways. The case of Juan, who successfully solved the problem using a graph rather than a formula, shows that the problem was not limited to algebraic solutions and that it could stimulate the student’s novelty. Thence, we can affirm that these tasks concerning algebraic computations for the students of our sample were true problems of algebraic modelling and that they were open to multiple solutions. According to the literature [26,28,29], the students’ exposure to different solving approaches and different representations is a teaching practice that can help them develop creativity.

Regarding RQ2—“in what ways can the use of multiple representations support the understanding of algebraic formulas?”—we saw that the association of algebraic formulas with their corresponding geometric measure supported the understanding of concepts and misconceptions. As examples, we can cite Excerpt 2, where students could understand why $\frac{a\cdot a}{2}$ is equal to $\frac{a^{2}}{2}$; and Excerpt 5, where the teacher used geometry to explain why a+a is not $a^{2}$. In line with the other previously cited results from the literature [17,18,19], the connection with geometry helped students visualize, manipulate, and understand variables, monomials, and polynomials. Moreover, the exploration of the formula in four different representation registers (geometric, algebraic, numeric, and graphic) was very useful to help clarify a difficult concept, such as the reason why $2a^{2}-\frac{1}{4}$ is not the three-quarters of the area of a rectangle (Excerpt 3). The translation in natural language added a fifth layer through which to interpret the formula. It was not one particular reasoning that made the students understand the concept, but the orchestration of different representations that was essential for understanding.

Concerning RQ3—“what are the main problems faced by students in using geometric thinking in geometry?”—we found the following:

- Fundamental misconceptions from arithmetic concepts, such as Mariana and Federico’s problems with fractions; and algebraic errors, such as *a* + *a* = *a*^2^
- Errors rooted in the comprehension of geometry, such as Marco’s recalling of the Pythagorean theorem to compute the triangle’s area.
- Problems coming from the well-established practice of studying the various mathematics branches separately, which lead students to stop reasoning algebraically when dealing with geometry.

At this stage of the experiment, the dialogues and discussions with the students participating in this experiment showed that the effects of a mnemonic method of teaching and learning Mathematics, based on routine exercises, rather than on the semantic aspects, were still visible. However, it seems that the activities had a positive effect in developing understanding and competence about algebraic computations. In fact, their results in the final test are surprisingly higher than the Italian average, if considering a specific item of the final test involving algebraic reasoning. We are aware that the sample considered in this experimentation is not a representative sample and that a quantitative comparison with the INVALSI data has some limitations; however, the difference in the results is surprising from a qualitative point of view also, given that the percentage of correct answers of students who followed the interactive path is near double the national percentage. We should point out that, in Italy, all lower secondary schools follow the same programs, and differences in students attending different schools depend only on the territorial context. The schools selected for our experiment belong to different socio-cultural contexts, from very low to upper ones.

The design of the activities presented in this paper follows all Kaput’s suggestions [30], as follows:

- We began early, since the activities involved students of grade eight, while algebraic manipulations will be formally developed in the following years. However, we need to point out that, although the study was aimed at grade eight students, activities of algebraic modelling can be introduced even earlier, from the primary level [31].
- The activities tried to apply algebra and integrate it in a geometry context, so to give a deeper meaning to the formulas and computations.
- They included different forms of algebraic thinking, in particular, considering Kaput’s framework [30], the syntactically guided manipulation of formalisms (when students were finding the formulas expressing the area of some figures), and the study of functions, relations, and joint variation (when the requests shifted to observe the growth of the area and display the function representing it).
- They are grounded in the students’ cognitive and, especially, linguistic abilities, since through the activities, attention was paid in translating from natural language to algebra as well as explaining and justifying students’ solutions.
- Active learning is encouraged through group work and digital interactive activities. In all the activities, students had the chance to explore and solve the problems themselves, and each group was asked to explain their solutions.

## 5. Conclusions

The activities discussed in this paper try to promote a shift from learning algebra as a set of technicisms to a functional approach to formulas and computations. This is attempted through two main aspects. First, by paying attention to the language and promoting dialogue on algebraic formulas. We saw in Excerpt 3, but also in the other excerpts, that the translation from natural language to algebraic language was successful and helped students focus on the structure of the formulas, understanding them from a syntactic viewpoint. Second, by underlying the role of the representations—as we could see in all the analyzed excerpts—every treatment was always explained by conversions in different semiotic registers, and the students’ activities were mostly based on conversions. The geometric register was prevalent to give meaning to algebraic formulas, but also the numeric approach and natural language were deeply used. This aspect is particularly relevant to develop full understanding of algebra at the school level. Moreover, as the results of this study show, the continuous shifts from one representation to another help students to develop creativity in problem solving and remain open to tackling problems from several perspectives. This is particularly useful in reducing the tendency of considering only one strategy as possible or correct and in training students to autonomously reason on each problematic situation, without following a default process mechanically, which may not be the easiest or most suitable one for a particular problem. The proposal of activities of this kind could have long-term results, such as the ability to integrate different kinds of reasoning while dealing with mathematical formulas and functions; moreover, it is crucially important to avoid the risk of losing this ability. Similar problem-solving activities can also be a way to activate an adaptive teaching model in the didactics [45,46]. Each student can feel free to choose their preferred strategy to solve a problem, and each strategy is legitimate. In further research, the connection between algebraic problem solving and adaptive teaching could be examined deeper.

From the analyzed literature and the results of this study, we can understand how teaching practices are important for developing algebraic knowledge, modelling skills, and creativity in problem solving, while avoiding misconceptions. Thus, sharing and spreading similar experiences and activities is fundamental. All the activities included in the interactive path were made available not only to the teachers involved in the project for their future classes but also to all the Italian teachers enrolled in the national “Problem Posing and Solving” project [47]. A training course for lower secondary school Mathematics teachers was proposed the following year [48]. The aim of the course was to help teachers to become autonomous in the design and management of similar classroom activities. Many of the teachers who experimented the activities in “Educating City” enrolled in this training course and continued to use similar activities in their didactics, having become aware of their effectiveness. It would be interesting to develop further the line of research concerning teacher training and algebraic modelling, in order to understand how a permanent change in the teachers’ practices affects the students’ understanding.

## Data Availability

The data presented in this study are available on request from the corresponding author. The data are not publicly available due to privacy reasons.
